# Discovery of Novel Rhabdoviruses in the Blood of Healthy Individuals from West Africa

**DOI:** 10.1371/journal.pntd.0003631

**Published:** 2015-03-17

**Authors:** Matthew H. Stremlau, Kristian G. Andersen, Onikepe A. Folarin, Jessica N. Grove, Ikponmwonsa Odia, Philomena E. Ehiane, Omowunmi Omoniwa, Omigie Omoregie, Pan-Pan Jiang, Nathan L. Yozwiak, Christian B. Matranga, Xiao Yang, Stephen K. Gire, Sarah Winnicki, Ridhi Tariyal, Stephen F. Schaffner, Peter O. Okokhere, Sylvanus Okogbenin, George O. Akpede, Danny A. Asogun, Dennis E. Agbonlahor, Peter J. Walker, Robert B. Tesh, Joshua Z. Levin, Robert F. Garry, Pardis C. Sabeti, Christian T. Happi

**Affiliations:** 1 FAS Center for Systems Biology, Department of Organismic and Evolutionary Biology, Harvard University, Cambridge, Massachusetts, United States of America; 2 Broad Institute, Cambridge, Massachusetts, United States of America; 3 Institute of Lassa Fever Research and Control, Irrua Specialist Teaching Hospital, Irrua, Edo State, Nigeria; 4 Department of Biological Sciences, College of Natural Sciences, Redeemer's University, Redemption City, Ogun State, Nigeria; 5 Department of Microbiology and Immunology, Tulane University, New Orleans, Louisiana, United States of America; 6 Lahor Research Laboratories and Medical Centre, Benin City, Edo State, Nigeria; 7 CSIRO Animal, Food and Health Sciences, Australian Animal Health Laboratory, Geelong, Australia; 8 Department of Pathology, University of Texas Medical Branch, Galveston, Texas, United States of America; 9 Department of Immunology and Infectious Disease, Harvard School of Public Health, Boston, Massachusetts, United States of America; The Global Alliance for Rabies Control, UNITED STATES

## Abstract

Next-generation sequencing (NGS) has the potential to transform the discovery of viruses causing unexplained acute febrile illness (UAFI) because it does not depend on culturing the pathogen or *a priori* knowledge of the pathogen’s nucleic acid sequence. More generally, it has the potential to elucidate the complete human virome, including viruses that cause no overt symptoms of disease, but may have unrecognized immunological or developmental consequences. We have used NGS to identify RNA viruses in the blood of 195 patients with UAFI and compared them with those found in 328 apparently healthy (i.e., no overt signs of illness) control individuals, all from communities in southeastern Nigeria. Among UAFI patients, we identified the presence of nucleic acids from several well-characterized pathogenic viruses, such as HIV-1, hepatitis, and Lassa virus. In our cohort of healthy individuals, however, we detected the nucleic acids of two novel rhabdoviruses. These viruses, which we call Ekpoma virus-1 (EKV-1) and Ekpoma virus-2 (EKV-2), are highly divergent, with little identity to each other or other known viruses. The most closely related rhabdoviruses are members of the genus *Tibrovirus* and Bas-Congo virus (BASV), which was recently identified in an individual with symptoms resembling hemorrhagic fever. Furthermore, by conducting a serosurvey of our study cohort, we find evidence for remarkably high exposure rates to the identified rhabdoviruses. The recent discoveries of novel rhabdoviruses by multiple research groups suggest that human infection with rhabdoviruses might be common. While the prevalence and clinical significance of these viruses are currently unknown, these viruses could have previously unrecognized impacts on human health; further research to understand the immunological and developmental impact of these viruses should be explored. More generally, the identification of similar novel viruses in individuals with and without overt symptoms of disease highlights the need for a broader understanding of the human virome as efforts for viral detection and discovery advance.

## Author Summary

Next-generation sequencing, a high-throughput method for sequencing DNA and RNA, has the potential to transform virus discovery because it does not depend on culturing the pathogen or *a priori* knowledge of the pathogen’s nucleic acid sequence. We used next-generation sequencing to identify RNA viruses present in the blood of patients with unexplained fever, as well as apparently healthy individuals in a peri-urban community in Nigeria. We found several well-characterized viruses in the blood of the febrile patients, including HIV-1, hepatitis B and C, as well as Lassa virus. We also discovered two novel rhabdoviruses in the blood of two apparently healthy (afebrile) females, which we named Ekpoma virus-1 and Ekpoma virus-2. Rhabdoviruses are distributed globally and include several human pathogens from the genera *lyssavirus* and *vesiculovirus* (*e*.*g*., rabies, Chandipura and vesicular stomatitis virus). The novel rhabdoviruses identified in this study are most similar to Bas-Congo virus, which was recently identified in an individual with an acute febrile illness. Furthermore, we demonstrate evidence of high levels of previous exposure to the two rhabdoviruses among our larger study population. Our results suggest that such rhabdovirus infections could be common, and may not necessarily cause overt disease. The identification of viral nucleic acid sequences in apparently healthy individuals highlights the need for a broader understanding of all viruses infecting humans as we increase efforts to identify viruses causing human disease.

## Introduction

Viral discovery is rapidly advancing, driven by the advent of high-throughput technologies like next-generation sequencing (NGS) [1]. Applying NGS as a diagnostic tool holds the promise for vastly expanding our understanding of the spectrum of microbes infecting humans, as it does not require *a priori* knowledge of the pathogens present. It also has the potential to elucidate the spectrum of disease-causing viruses in patients with undiagnosed acute febrile illness (UAFI), a common occurrence in health clinics around the world [2]. NGS can also serve to increase the power of surveillance systems to detect infrequent zoonotic transmissions that have the potential to become pandemics [3].

NGS has already been used successfully as both a diagnostic tool and a means to discover novel viruses associated with human disease [4–8]. Examples of these discoveries include novel arenaviruses [5], phleboviruses [4], and coronaviruses [8]. Recently a novel rhabdovirus, now referred to as Bas-Congo virus (BASV), was identified in the blood of a patient from central Africa who was suspected of suffering from viral hemorrhagic fever [9].

However, a better understanding of the spectrum of viruses infecting humans is needed to fully realize the potential of NGS and differentiate between pathogenic and non-pathogenic viruses. This global problem is particularly acute in tropical regions throughout the world, where the burden of infectious disease remains high and the bloodstream virome of large numbers of apparently healthy individuals has not been characterized. Most studies of UAFI lack comparisons with apparently healthy individuals and rely on small-scale associations (in some cases even a single patient sample) without any statistical support or the ability to determine causality [7,9].

In this study we use high-throughput NGS to elucidate the spectrum of RNA viruses present in the blood of patients with UAFI in a population from southeastern Nigeria, using apparently healthy members of the same community for comparison. While we detected only known and common viral nucleic acid sequences in the UAFI patients, we were able to assemble full-length genomes of two novel, highly divergent rhabdoviruses from two apparently healthy individuals. We found that these viruses were similar to BASV and to viruses of the genus *Tibrovirus*. By conducting a serosurvey of our study cohort, we found that exposure to these novel viruses was unexpectedly high. Our findings suggest that human infection with certain types of rhabdoviruses may be common, and highlight the need for a broader understanding of the human virome as the use of NGS for microbial discovery advances.

## Materials and Methods

### Study population

Our study population consisted of men and women from all age groups and socioeconomic backgrounds living in and around Irrua, a modest-sized peri-urban village in southeastern Nigeria (for further descriptions of the study population see S1 Table). As part of a partnership with the Irrua Specialist Teaching Hospital (ISTH) to study Lassa fever, we collected blood samples from suspected Lassa fever patients that tested negative for Lassa virus by reverse transcription PCR (RT-PCR) and subjected them to NGS (S1 Table). We hypothesized that UAFI patients with symptoms resembling viral hemorrhagic fever could be infected with other pathogens that cause severe illness. We additionally collected samples from apparently healthy individuals (*i*.*e*., individuals whose temperature was in the normal range and did not have any overt symptoms of illness) from the surrounding populations as part of the 1000 Genomes Project, and as part of a control population for our studies of Lassa fever.

### Ethics statement

We performed collections of febrile cases and apparently healthy controls under approved IRB protocols in Nigeria (Oyo State Ministry of Health, ISTH) and the US (Tulane University, Harvard University, Harvard School of Pubic Health, and the Broad Institute). All adult subjects provided informed consent, and a parent or guardian of any child participant (aged under 18 years) provided informed consent on their behalf. All children 7 and older additionally provided assent. Individuals provided written informed consent. If an individual was unable to read, a study staff read the document to the participant or guardian. The individual then provided a thumbprint, and the consent form was cosigned by the study staff as well as a witness. The use of thumbprints was specifically approved by the IRB granting institutions.

### Sample collection

We collected approximately 5–10 mL of venous blood in EDTA vacutainer tubes, centrifuged them to obtain the plasma from cellular fractions, and inactivated the plasma by adding buffer AVL (Qiagen). We added carrier RNA to some of the samples as indicated in S2 Table. In the case of the apparently healthy controls, we collected an additional aliquot of ‘unadulterated’ plasma that was not inactivated with buffer AVL.

### RNA-seq library construction of UAFI samples

We constructed RNA-seq libraries as previously described [10]. We prepared some of the libraries from extracted RNA for either single individuals (referred to as singletons) or from RNA pooled from several individuals (referred to as pools) (S2 Table). We treated all samples with DNase. We primed RNA using random hexamers, or modified hexamers (5’-NNNNNNV-3’ from Integrated DNA Technologies) if carrier RNA was present in the sample. We amplified the resulting libraries by PCR, pooled, and sequenced on an Illumina HiSeq 2500 according to the manufacturer’s specifications. Primers used for Sanger sequencing are listed in S3 Table. The raw data has been deposited to SRA under BioProject ID PRJNA271229.

### RNA-seq library construction of healthy controls

We processed individual afebrile controls as described for UAFI samples; however, the method of pooling differed. We pooled and filtered unadulterated plasma (without AVL) samples and centrifuged them at 104,000 x *g* for 2 hours at 4°C. We resuspended the viral pellet in buffer and used it to construct libraries for sequencing. AVL denatures viral particles, thus preventing centrifugation of the particles. We have observed comparable results between samples inactivated by AVL and those that are not.

### Bioinformatics pipeline to identify viruses

We trimmed raw Illumina sequences consisting of 100 bp paired-end reads to remove bases from the ends of the reads with low quality scores, and discarded all reads shorter than 70 bp after quality trimming. We removed human and other contaminating reads using BMTagger (NCBI), and removed duplicate reads and low complexity reads using PRINSEQ [11]. We assembled reads *de novo* using MetaVelvet [12] followed by Trinity [13]. We used contigs of at least 200 bp for BLASTn or BLASTx queries of the GenBank nucleotide (NT) or protein (NR) databases (E-score cutoffs of 10^-6^ and 10^2^, respectively). In a parallel pipeline, we used individual reads for BLASTn or BLASTx queries of GenBank with the same E-score cutoff values. We performed taxonomic classification of assembled contigs and individual reads and visualized them using MEGAN 4 [14]. We considered samples to have a virus present if MEGAN 4 ‘min support’ was ≥5 and ‘min score’ was ≥50. We assessed statistical significant differences in the distributions of viruses between UAFI samples and apparently healthy individuals using a two-tailed Fisher’s exact test with α<0.05 considered significant.

### Quantitative PCR of viral copy number

We used quantitative real-time PCR to measure the number of Ekpoma viral RNA copies per milliliter of blood using the RNA-to-C_T_ 1-Step Kit (Applied Biosystems). The primers, which amplify an ~100bp region in the polymerase (L) gene, have the following sequences:: EKV-1: 5’-AAGAGTTGTTGGGATGGTCAGA-3’ (forward) and 5’- TGATTCTTGCTTCTCGCTCGAT-3’ (reverse); and EKV-2 primers: 5’-TGGCCAATTCCTTGGCTATCCCCT-3’ (forward) and 5’-TCCCGCCGGAGACATACATCTT-3’ (reverse). We amplified PCR reactions on the ABI 7900 sequence detection system using the following cycling parameters: 30 minutes at 48°C, 10 minutes at 95°C, and 40 cycles of 15 seconds at 95°C and 1 minute at 60°C. A serial dilution of a synthetic DNA amplicon, which corresponds to the amplified region of the polymerase gene, was used to quantify the number of viral cDNA copies in the reaction. Human K562 RNA and RNA purified from the plasma of an afebrile individual (244M), were used as negative controls.

### Phylogenetic analysis

We performed multiple sequence alignments of rhabdovirus nucleoprotein (N), glycoprotein (G), matrix (M), phospoprotein (P) and polymerase (L) amino acid sequences using MAFFT v6.902b18 [15] with the following parameters:—localpair—maxiterate 1000—reorder—ep 0.123 before being trimmed using trimAl v1.419 [16] with the maximum likelihood specific parameter:-automated1. We used PROTTEST [17] to identify rtREV+I+G [18] as the best evolutionary model and made maximum likelihood phylogenies with RAxML v7.3.0 [19]. Trees were bootstrapped using 500 pseudo-replicates. We also created trees using MrBayes v3.2 [20]. We first built trees using 46 rhabdovirus sequences and included parainfluenza virus-1 as an outgroup, to find the novirhabdoviruses as the likely root of the rhabdovirus tree, which has been previously described [21]. We then excluded parainfluenza virus-1 and built a tree using the 46 rhabdovirus sequences (S6A Fig), which allowed us to select VSV as a likely outgroup for the tibroviruses and ephemeroviruses. Subsequent alignments and trees were then created using only the tibroviruses and ephemeroviruses, including EKV-1, EKV-2, and BASV, as well as VSV. We found that using parainfluenza virus-1 or the novirhabdoviruses as the root, gave the same tree topology. Relevant accession numbers can be found in S4 Table.

### Serosurvey for EKV-1 and EKV-2

We cloned His-tagged N genes from EKV-1 and EKV-2 into pET45B(+) and expressed them in *E*. *coli*. We lysed the cells in the presence of protease inhibitors and purified the proteins with HisPur Ni-NTA Spin Columns (Thermo Scientific). We confirmed the purity of the proteins by Western Blot. We created ELISA plates by coating the EKV-1 and EKV-2 N proteins onto 96-well plates at 2μg/mL in carbonate-bicarbonate buffer overnight at 4°C. Human IgG specific to EKV-1 or EKV-2 was detected by ELISA as previously described [22]. We calculated cut-off values based on the mean of the US controls (N = 137) plus three or five standard deviations.

## Results

### Clinical characteristics of study subjects

We selected blood samples from 195 UAFI and 328 afebrile controls for RNA sequencing by Illumina NGS (S1 Fig). We collected a number of demographic and clinical parameters (S1 Table) for each individual in our study.

### Illumina NGS sequencing of more than five hundred human blood samples

We successfully constructed 120 RNA-seq libraries from UAFI samples (94 singletons and 26 pools) comprising a total of 195 individuals, and 58 RNA-seq libraries from afebrile apparently healthy control samples (34 singletons and 24 pools) comprising a total of 328 individuals (S5 Table). Illumina sequencing generated a total of 3.71 billion 100 base pair (bp) paired-end reads. We analyzed these samples using a bioinformatics and computational pipeline developed in our laboratory (S2A Fig). After filtering out low-quality sequences, duplicates, human reads and common contaminants, less than 0.5% of the reads typically remained in each library (S2B–D Fig).

### Viral sequences in UAFI patients correspond to known pathogens

**Fig 1:**
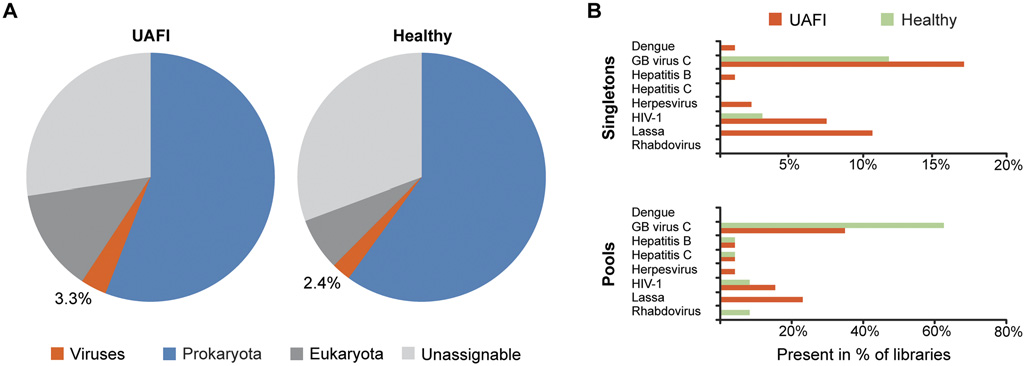
Viruses identified in UAFI samples and afebrile controls. (**A**) Overview of reads we identified in Illumina HiSeq reads from 94 singleton UAFI samples and 34 apparently healthy singleton control samples. For all samples, we removed human reads and common bacterial contaminants, and subjected the remaining reads to BLASTn and BLASTx queries of GenBank and assigned to taxonomic kingdoms using MEGAN 4. (**B**) Viruses identified in singleton (RNA-seq libraries constructed from a single individual) and pooled libraries (RNA-seq libraries constructed from several individuals). In the case of pooled libraries, the percentage refers to the number of libraries, not individual samples.

We examined the overall composition of reads identified in 94 singleton UAFI samples and in 34 apparently healthy singleton controls (Fig. 1). We found ~25% of the filtered reads returned no BLAST hit or were unable to be unequivocally assigned to the eukarotya, prokaryota or viral kingdoms. The majority of filtered reads in both UAFI and afebrile libraries were bacterial.

The overall percentage of viral reads was similar between UAFI patients and afebrile controls (3.3% and 2.4%, respectively). The majority of viral reads were derived from three sources: human adenovirus C, phages, or GB virus C (S6 Table and S1 Text). GB virus C, a non-pathogenic RNA virus [23], was identified in 18% of UAFI singleton libraries and 12% of singleton healthy controls (Fig. 1B and S3 Fig); a higher percentage of pooled healthy controls contained GB virus C, possibly because each pool contained a greater number of individual samples compared to the UAFI pools. We identified several well-characterized pathogenic RNA viruses, including LASV, HIV-1, hepatitis C and dengue virus in the UAFI patients (Fig. 1B and S6 Table). We did not find any evidence for the presence of Ebola virus. LASV was the most frequent pathogenic virus observed in UAFI cases and the only virus statistically enriched in the UAFI as compared to the apparently healthy controls (*P*-value = 0.002, Fisher’s test; S3 Fig). Although samples were DNAse treated, we also detected several DNA viruses, including hepatitis B virus, herpesvirus 4 (Epstein-Barr virus), herpesvirus 5 (human cytomegalovirus), and herpesvirus 8 (Kaposi’s sarcoma virus) (Fig. 1B and S6 Table).

### Discovery of two novel rhabdovirus sequences in afebrile controls

In two pools of RNA from afebrile individuals, we identified reads with distant relationships to previously identified rhabdoviruses. A PCR assay developed to identify the infected individual within each pool revealed two infected females aged 45 (sample 13M) and 19 (sample 49C). We named the two viruses Ekpoma virus-1 (EKV-1; from 13M) and Ekpoma virus-2 (EKV-2; from 49C) because both individuals lived in Ekpoma, a village located about ten kilometers from ISTH.

**Fig 2:**
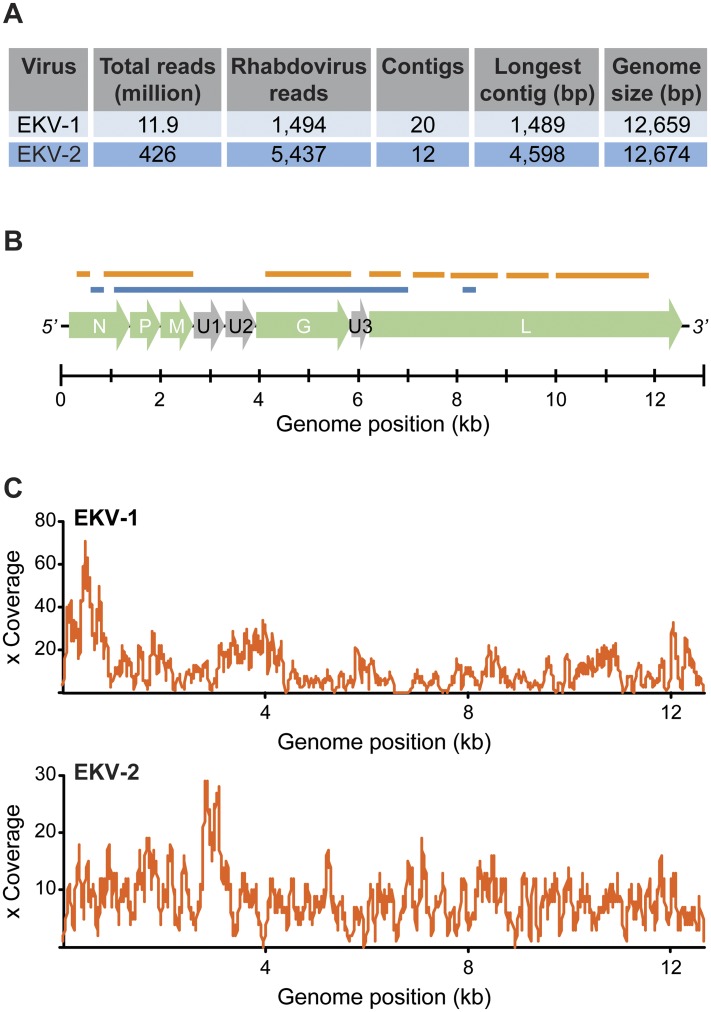
Sequencing results and schematic representation of the EKV-1 and -2 genome organization. (**A**) Overview of the data generated for each novel rhabdovirus. (**B**) A schematic showing the assembled genomes, consisting of the following genes: *nucleoprotein* (N), *phosphoprotein* (P), *matrix* (M), *U1*/*U2*/*U3* (uncharacterized accessory proteins), *glycoprotein* (G), and *polymerase* (L). We indicate in orange (EKV-1) and blue (EKV-2) segments of the viral genomes that could not be assembled from Illumina reads and instead Sanger sequenced. (**C**) Coverage plots of the final viral genomes.

We assembled several long contiguous overlapping rhabdovirus sequences (contigs) (Fig. 2A). From these contigs we synthesized virus-specific primers for EKV-1 and EKV-2 and used Sanger sequencing to confirm the results of Illumina sequencing and fill in missing parts of the genomes (Fig. 2B). The combined sequencing produced two genomes of 12,659 bp (EKV-1) and 12,674 bp (EKV-2) (GenBank accession numbers KP324827 and KP324828).

The coverage of EKV-1 ranged from 1–71x (median 9x) and the coverage of EKV-2 ranged from 1–29x (median 8x; Fig. 2C). We did not find any additional samples that contained reads from these two novel rhabdoviruses.

### EKV-1, EKV-2, and BASV cluster within the genus *Tibrovirus*

The *Rhabdoviridae* family includes at least eleven genera [24]. We found that the genomic organization of EKV-1 and EKV-2, like BASV, is the same as members of the genus *Tibrovirus* (S4 Fig). The viral genomes consist of the prototypical five open reading frames (ORFs) found in most rhabdoviruses (N, P, M, G, and L) as well as at least three additional ORFs of unknown function (U1 to U3) [25] (Fig. 2B). The latter three ORFs are also seen in other members of the genus *Tibrovirus* and their presence clearly distinguishes these viruses from the closely related genus *Ephemerovirus*.

We found that the sequence identity among the Ekpoma viruses was low, ranging from 33.2–39.4% for the different ORFs at the protein level (S4 Fig). The nucleoprotein and polymerase were the most highly conserved proteins (S5 Fig), while U1–U3 were the most divergent. Overall, EKV-2 was more similar at the amino acid level to BASV (39.4% identity) than it was to EKV-1 (35.1% identity).

**Fig 3:**
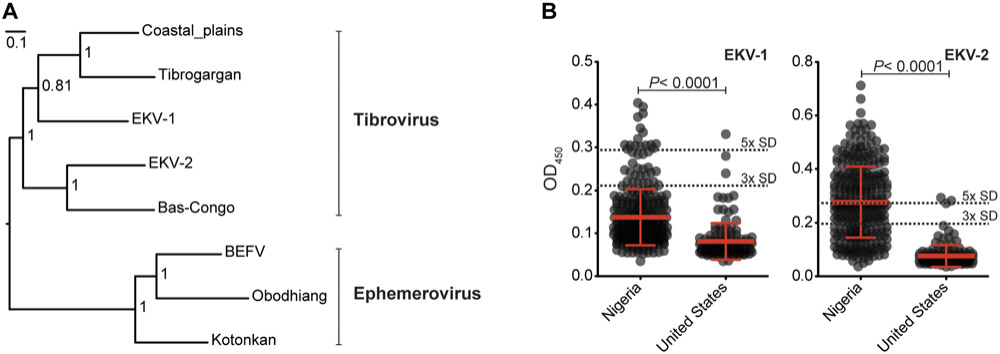
Phylogenetic analysis of rhabdovirus polymerase proteins. We created a Bayesian phylogenetic tree using full-length polymerase (L) proteins obtained from GenBank. (**A**) Tree based on alignments of the tibroviruses and ephemeroviruses. Posterior probabilities are shown at relevant nodes (scale bar = nucleotide substitutions/site) and the tree was midpoint rooted. (**B**) ELISA detection of EKV-1 and EKV-2 IgG in cohorts from Nigeria and the United States (US normals). Each circle correspond to the raw OD_450_ value of an individual sample. The mean +/- standard deviation (SD) is shown. Black dotted line = cut-off values for positive samples based on the mean of US normals plus 5x or 3x SD. *P*-values were calculated using a two-tailed Mann-Whitney test.

To determine the place of the Ekpoma viruses within the rhabdovirus phylogeny we constructed maximum likelihood and Bayesian trees for the major viral proteins. We found that EKV-1 and EKV-2 clustered with BASV, TIBV, and Coastal Plains virus (Figs. 3A and S6). We further found that EKV-1 is a closer evolutionary relative to TIBV than to EKV-2 or BASV. EKV-2, in contrast, formed another branch with BASV (Fig. 3A, B). Though these viruses were discovered in geographically distant locations, phylogenetic analyses suggest the presence of a distinct group of viruses in the *Tibrovirus* genus capable of human infection. Based on phylogenetic relationships, host range and genomic architecture, we propose that BASV, EKV-1 and EKV-2 should all be included within the genus *Tibrovirus*.

### High human exposure rates to rhabdoviruses in Nigeria

To assess the level of human exposure to the novel rhabdoviruses, we developed enzyme-linked immunosorbent assays (ELISAs) to detect antibodies that recognized the N proteins of EKV-1 and EKV-2. We performed a serosurvey for EKV-1 and EKV-2 on 457 samples and found that significantly more Nigerian individuals (n = 320) had EKV-1- and EKV-2-specific antibodies than apparently healthy US controls (n = 137; Fig. 3C; *P*-value < 0.0001, Mann-Whitney test). Using conservative positivity cut-off values, we found that ~10% of Nigerian individuals show evidence of previous exposure to EKV-1 (Table 1 and Fig. 3C). The seropositivity to EKV-2 was much higher, with ~50% of Nigerians showing evidence of previous exposure (Table 1 and Fig. 3C). We did not observe any significant difference in the sex or age-range of the individuals with antibody titers to EKV-1 or EKV-2 (S7 Fig). We cannot rule out that our assays do not cross-react with other similar rhabdoviruses, which could inflate the overall seroprevalence observed for the Ekpoma viruses; however, it should be noted that limited cross-reactivity was observed between EKV-1 and EKV-2 (S8A Fig). While we found strong cross-reactivity between our assays for EKV-1 and rabies virus (S8B Fig), the correlation between EKV-2 and rabies virus was much less pronounced (S8C Fig). Importantly, when testing general cross-reactivity in our assays by comparing the ELISA results from the rhabdoviruses to that of LASV, we did not find any correlations (S8D–F Fig).

**Table 1:** Sero-positivity to EKV-1 and EKV-2.

	*3x SD cut-off*		*5x SD cut-off*	
	EKV-1	EKV-2	EKV-1	EKV-2
Nigeria	12%	69%	5%	45%
United States	2%	2%	1%	1%

A serosurvey for EKV-1 and EKV-2 was performed on Nigerian samples (n = 320). Cut-off values were based on the mean of US normals (n = 137) plus either 3xSD or 5xSD (SD = standard deviation).

Acute infection with RNA viruses often produces high viral loads. To assess the level of viremia, we used quantitative real-time PCR to measure EKV-1 and EKV-2 viral copy number. We detected 4.5 million viral genome copies per milliliter of plasma in the individual infected with EKV-1 and 46,000 viral genome copies per milliliter of plasma in the individual infected with EKV-2 (S9 Fig). These numbers, while informative, should be interpreted with caution, as sample degradation may have affected the number of viral copies detected.

### Follow-ups with EKV-1 and EKV-2 infected individuals

After the discovery of the two Ekpoma viruses, we sought to further determine the health of the infected individuals. Nearly two years after their initial blood draw, we conducted oral interviews with both individuals and collected convalescent serum samples. Both individuals tested negative for the two Ekpoma viruses by PCR upon testing of their convalescent samples (S10 Fig); however, using our ELISA assays, we found that they both had antibodies reacting with EKV-1 or EKV-2, as expected (S11 Fig). Notably, while both individuals had antibody titers at the time of infection and in the follow-up samples, the woman infected with EKV-2 showed lower titer in her follow-up sample, as compared to the original blood draw (S11B Fig).

The woman infected with EKV-1 could not recall any episode of febrile illness in the weeks or months following the collection of her initial blood sample. The woman infected with EKV-2 revealed that she suffered an episode of febrile illness two weeks after we collected her blood sample. She was admitted to the hospital where her illness was clinically diagnosed as malaria. While the individual’s illness resolved after anti-malarial treatment, we cannot confirm whether a malaria parasite was the causal agent.

### Culture and attempted isolation of Ekpoma rhabdoviruses

We attempted to isolate EKV-1 and EKV-2 by using plasma from the infected individuals to inoculate cultures of Vero E6, BHK, C6/36 mosquito, LLC-MK_2_, SW13 and biting midge (*Culicoides variipennis*) cell lines. We did not observe any evidence of viral cytopathic effects in these cultures, nor could we detect any virus by qPCR or electron microscopy. We also attempted to isolate the viruses by intracranial inoculation of newborn mice; however, we did not observe any signs of illness over 14 days. It is possible that the viruses may not be able to infect any of the tested cells or animals, however, potential sample degradation may have compromised the infectivity of viral particles.

## Discussion

We used high-throughput NGS to elucidate the spectrum of RNA viruses present in the blood of patients with UAFI in a population from southeastern Nigeria, using apparently healthy members of the same community for comparison. NGS has the advantage of being able to identify pathogens without culturing or *a priori* knowledge of the pathogen’s nucleic acid sequence.

Despite the advantages of NGS, there are certain biases in our approach. First, the selection of blood limited our investigation to a single anatomical compartment. Many viruses cannot be detected in the blood (*e*.*g*., rabies virus which is strictly neurotropic). A complete understanding of a febrile or healthy person’s virome necessitates sequencing of all tissues in the body, which for practical reasons, is not possible. The ability to identify novel viruses is also limited to sequences that have some homology existing sequences in a public database. Highly divergent and truly novel pathogens may be missed by conventional BLAST searches. In our study, ~25% of filtered reads returned no BLAST hit or were unable to be unequivocally assigned to the eukaryotya, prokaryota or viral kingdoms. Despite these limitations however, we were able to identify EKV-1 and EKV-2, both of which have only about 35% amino acid similarity to already known viruses.

In our study we made an unexpected discovery of nucleic acid sequences suggestive of novel rhabdoviruses in our apparently healthy controls. The identified viruses, EKV-1 and EKV-2, most closely resemble members of the genus *Tibrovirus*, and in particular BASV, based on genomic structure and phylogenic analyses. BASV was recently identified in an individual from central Africa displaying symptoms suggestive of viral hemorrhagic fever [9]. Despite detection in an apparently healthy individual, EKV-2 is the most closely related virus to BASV identified to date.

**Fig 4:**
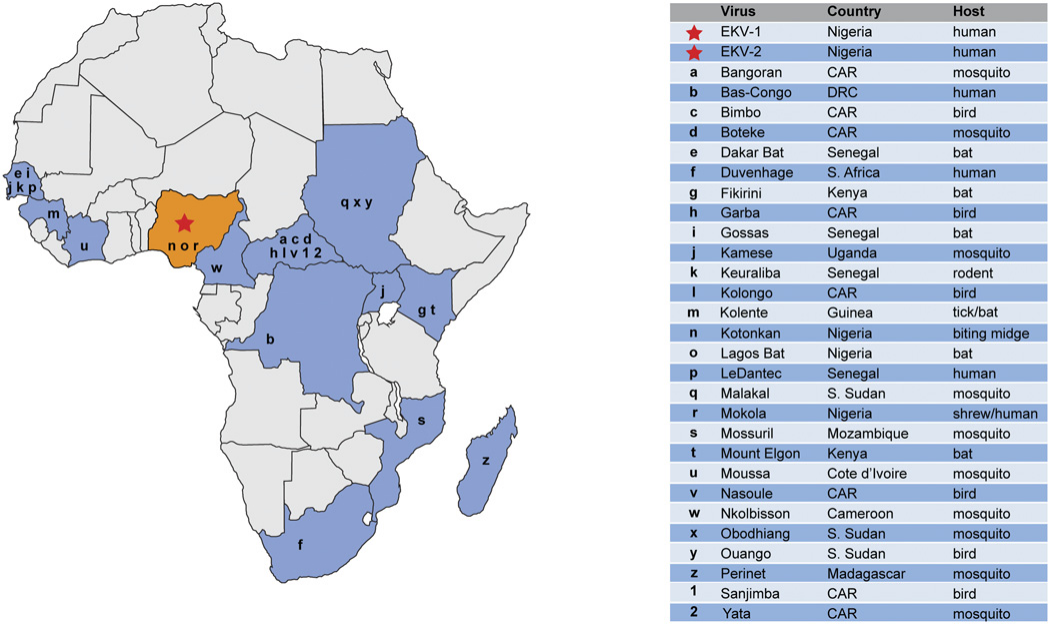
Examples of rhabdoviruses reported in Africa. A map depicting examples of rhabdoviruses isolated in sub-Saharan Africa. This map does not depict the current distribution of rhabdoviruses in Sub-Saharan Africa, nor is it meant as a comprehensive listing of all rhabdoviruses isolated in Africa; rather its purpose is to illustrate that many rhabdoviruses have been discovered throughout Africa over the past half-century. Country refers to the sample’s country of origin. Abbreviations: CAR, Central African Republic; DRC, Democratic Republic of Congo.

Tibroviruses, including Tibrogargan, Coastal plains and Bivens Arm viruses, are transmitted by *culicoidies* insects and are known to cause subclinical infections in cattle and other ruminants [26]. Their amino acid sequence similarity to Tibrogargan and Coastal Plains viruses raises the possibility that they might be vector-borne [26–29]. If true, infection could be common in environments where biting insects are ubiquitous, like central and western Africa. Many rhabdoviruses have already been discovered in sub-Saharan Africa using conventional methods—mostly in insects and vertebrates (Fig. 4). Our results suggest many more remain to be discovered, and that a number of these may infect humans.

Consistent with the potential for widespread and subclinical infection by rhabdoviruses, our serosurvey uncovered evidence for very high exposure to EKV-1 or EKV-2, with nearly 50% of our apparently healthy cohort showing evidence of EKV-2 exposure. Despite this high rate, we did not detect any EKV-1 or EKV-2 nucleic acids in the UAFI patients. These results suggest that members of the genus *Tibrovirus* are unlikely to be common causes of viral hemorrhagic fever as has been suggested for BASV [9].

We attempted to isolate EKV-1 and EKV-2, but were unsuccessful in our efforts. We speculate that sample handling may have caused degradation of viral particles. Alternatively, these novel viruses may not infect the common cell types we selected for culturing. Historically, isolating a virus from an infected individual is a necessary step for demonstrating the existence of the novel virus and that the patient was infected. However, as NGS becomes more common, it is likely that many new viruses will be identified that cannot easily be cultured. That does not mean these viruses cannot be studied biochemically or “recreated” in the laboratory. Parts of the virus can be synthesized *de novo* and incorporated into existing viral vectors. In some cases, the entire nucleic acid sequence of the virus can be synthesized *de novo*, introduced into cells, and potentially cultured.

The recent discovery of three related rhabdoviruses—two in apparently healthy individuals (this study) and one in an acutely ill patient [9]—highlights the challenges of determining the true cause of unexplained illness. Many factors determine whether a particular virus will produce disease in the infected host, including genetic variation in the virus and the host, nutritional and immune status, and the presence of co-infections that may increase susceptibility to otherwise benign agents. Identifying the cause of disease becomes even more challenging since multiple microbes are present in a sample, including commensal bacteria and viruses.

Proving disease causality is a centuries-old problem and identifying a potential pathogen is merely the first step in a long process. Researchers have recently proposed revisions to Koch’s postulates—the first framework for assessing causality—in light of advancing modern molecular techniques [30,31] to add rigor to the pursuit. Yet there are still a number of limitations to current studies. For many studies, investigators were only able to study a single patient sample [9]. Without sufficient numbers of samples from infected patients and matched apparently healthy individuals, it is impossible to interpret the clinical significance of a single virus detection. It remains possible that BASV produced an asymptomatic infection, like the control subjects infected with EKV-1 and -2 in our study, while the acute illness was actually due to another agent, like the rotavirus (which the authors propose was a laboratory contaminant), or one of the many bacteria also present in the sample [9]. Of course, the true source of the infection could have been none of the microbes identified in the blood. Sampling of other tissues would be needed to rule out localized infections as the cause of disease.

Regardless of whether infection with particular rhabdoviruses is symptomatic or not, the discovery of novel rhabdoviruses could be of importance to human health. Members of the *Rhabdoviridae*, such as lyssaviruses and vesiculoviruses, produce serious neurotropic disease in humans [32,33]. Others, such as vesicular stomatitis virus (VSV), produce subtle neurotropic infections with few acute disease symptoms. BASV, like VSV, appears to have broad tissue tropism [34] and may infect similar cell types. Further studies are needed to determine if the novel rhabdoviruses discovered in this study produce neurotropic outcomes in humans similar to those of lyssaviruses and vesiculoviruses [35–37].

How should future studies using NGS tackle the issue of disease causality in these and other newly discovered microbes? The most obvious approach involves finding a statistical association with the microbe in disease and non-disease states, similarly to what we show for LASV in this study (S3 Fig). This requires collecting matched controls from either the patient or members of the community who do not have the disease. This approach faces its own challenges. If viral or host factors play a substantial role in disease outcome, it might necessitate large sample collections. Isolation of the pathogen and propagation in an animal model or tissue culture can provide valuable insights into its pathogenicity and effect on the host’s response to infection.

The recent advent of NGS has the potential to transform the centuries-old pursuit of finding disease-causing pathogens and to elucidate the complete human virome. But in the process, it will be important to be cautious. As the vast majority of viruses studied over the past century have been those that cause disease, the large-scale sequencing of samples from vertebrates and insects will likely be biased towards identifying novel benign viruses rather than pathogenic ones. Although many newly discovered viruses may not cause overt symptoms of disease, they may have immunological and developmental consequences—perhaps by increasing susceptibility to other pathogens or affecting other aspects of human development.

Pathogen discovery tools are evolving rapidly. Investigations that harness these new tools will likely identify a plethora of new viruses in humans, animals, and insects. Developing systems to assess causality, especially through the thorough sampling of non-disease-affected controls, will be critical to realizing the potential of NGS as a routine diagnostic tool.

## Supporting Information

S1 Fig Clinical characteristics of study population. The age and gender of UAFI patients and healthy controls is indicated in the top table. Clinical symptoms of UAFI patients are described in the bar graph. The attending physician observed and recorded symptoms were at the time the patient was admitted to the hospital.
(PDF)

S2 Fig Overview over the informatics pipeline. (**A**) General overview over the various steps in the computational pipeline. (**B-D**) Plots over the various depletion steps performed in the informatics pipeline from three representative samples containing the EKV rhabdoviruses (left panel = overall reads retained after each step, right panel = % reads removed in each step compared to the previous step). (**B**) Sample 49CMiSeq (singleton, MiSeq, EKV-2). (**C**) Sample 49CHiSeq (singleton, HiSeq, EKV-2). (**D**) Sample HP1_LIB11–18 (pool, MiSeq, EKV-1).
(PDF)

S3 Fig Comparison of viruses identified in UAFI and healthy individuals. The distributions of the major viruses identified in UAFI patients and healthy individuals were compared using a two-tailed Fisher’s exact test (ns = not significant). LASV was the only virus shown to be significantly enriched in UAFI patients. No viruses were found to be significantly enriched in the healthy individuals. Only the results for LASV, GB virus C and the Ekpoma rhabdoviruses are shown.
(PDF)

S4 Fig Protein similarity plots of EKV-1, -2, BASV and TIBV. We generated similarity plots by aligning concatenated amino acid sequences and calculating scanning amino acid pairwise identities using a 50 bp window. The x-axis represents the amino acid position along the concatenated rhabdovirus amino acid sequence and the y-axis represent percent pairwise similarity. The percent identity of each pairwise comparison for the individual genes is shown beneath each plot (dashed grey line = 50% identity; red blocks = less than 30% identity).
(PDF)

S5 Fig Amino acid alignment of the nucleoprotein from EKV-1, -2, BASV and TIBV. We aligned complete nucleoprotein amino acid sequences from the indicated rhabdoviruses using MAFFT. A complete nucleoprotein sequence for BASV is not available. Residues colored green represent identical amino acids in all four viruses; residues colored yellow represent identical amino acids in three of the four viruses. The overall pairwise identity for each set of compared viruses is shown in the table.
(PDF)

S6 Fig Phylogenetic analysis of rhabdovirus N, G, M, and P proteins. We created Bayesian and maximum likelihood phylogenetic trees using full-length proteins obtained from GenBank. (**A**) Bayesian tree of full-length polymerase (L) proteins based on alignments from all obtained rhabdovirus sequences. The tree was rooted using the novirhabdovirus clade and posterior support values are shown for key nodes. (**B-F**) Trees based on alignments of the tibroviruses and ephemeroviruses using vesicular stomatitis virus as an outgroup. (**B**) L proteins, (**C**) M proteins, (**D**) P proteins, (**E**) N proteins, and (**F**) G proteins. Bootstrap support values and posterior support are shown for each node (500 pseudo-replicates). Trees were rooted using vesicular stomatitis virus. Scale bar = nucleotide substitutions/site.
(PDF)

S7 Fig Age and gender distribution of sero-positivity to EKV-1 and EKV-2. **(A, B)** Box plots showing the mean and the min to max raw OD_450_ values obtained from IgG ELISAs specific for EKV-1 and EKV-2. **(A)** Gender distribution. **(B)** Samples were grouped into bins of individuals younger than 30 years old or 30 years and older. **(A, B)** Distributions were compared using a Mann-Whitney test, but no statistical significant differences were observed among the groups.
(PDF)

S8 Fig Correlation between seropositivity to EKV-1 and EKV-2. OD_450_ values obtained from IgG ELISA assays specific for EKV-1, EKV-2, LASV, and rabies virus (RABV) were normalized by comparison to a calibration series run on each plate and plotted against each other. r = Pearson correlation coefficient.
(PDF)

S9 Fig Quantitative real-time PCR analysis of EKV-1 and -2 viral RNA copy number. We determined viral copy number using RNA extracted from plasma, primers that target the polymerase (L) gene and serial dilutions of a synthetic amplicon corresponding to the amplified target. We repeated each PCR experiment three times independently. Total human RNA purified from K562 from leukocytes and RNA purified from 244M, the plasma of an afebrile control, were used as a controls.
(PDF)

S10 Fig PCR for EKV-1 and EKV-2 on original and follow-up samples. We performed reverse transcription followed by PCR on RNA extracted from the original plasma samples and follow up plasma samples and electrophoresed on a 2.2% agarose gel with ethidium bromide. Primer sets were specific for either EKV-1 or EKV-2.
(PDF)

S11 Fig ELISAs specific for immunoglobulin G antibodies against EKV-1 and EKV-2 on original and follow-up samples. We compared a dilution series of the original sample to the follow-up sample for 13M and 49C with α-His tag IgG as a positive control. **(A)** Raw OD_450_ values for samples from patient 13M on EKV-1 NP-coated ELISA plate. **(B)** Raw OD_450_ values for samples from patient 49C on EKV-2 NP-coated ELISA plate.
(PDF)

S1 Table Clinical and demographic characteristics of study population. (XLSX)

S2 Table Sample processing and RNA-seq library construction methods. For extraction method, ‘direct’ refers to RNA extracted directly from plasma using the QIAamp Viral RNA Extraction Kit and ‘ultracentrifugation’ refers to plasma that is pooled and subjected to ultracentrifugation before RNA is extracted from the resulting pellet. RT method refers to cDNA synthesis and carrier RNA removal: (1) ‘random’ refers to samples without carrier RNA in which RNA was primed with random hexamers; (2) ‘mod-hex’ refers to samples with carrier RNA in which RNA was primed with a modified-hexamer as described in Materials and Methods; (3) ‘random-PP’ refers to samples with carrier RNA that in which the RNA was primed using random hexamers and then carrier RNA removed (after cDNA synthesis) using Pippin Prep size selection.
(XLSX)

S3 Table Sanger sequencing primers. A list of the primers used for Sanger sequencing of EKV-1 and -2.
(XLSX)

S4 Table Accession numbers of viral genomes used for phylogenetic analysis. (XLSX)

S5 Table Overview of the sequencing libraries created in this study. (DOCX)

S6 Table Viruses identified in UAFI and afebrile samples. This spreadsheet has 3 parts: (**Tab 1**) a table of the major viruses identified with at least 5 reads per library and minimum MEGAN score of 50 (BLAST columns), including the number of reads that realigned to viral genomes assembled from this study (Novoalign column) and the overall homology of genomes that could be assembled as compared to their closest relative in GenBank is indicated in Column H; (**Tab 2**) a complete list of all viral reads identified by BLASTn and BLASTx GenBank queries and taxonomically assigned using MEGAN 4 (note that in this table the BLASTn and BLASTx reads have been combined into a single value); (**Tab 3**) A list of viral genomes assembled from the RNA-seq libraries using Trinity and used for realignment of reads using Novoalign.
(XLSX)

S1 Text Additional information about the identified viruses. (PDF)

## References

[1] Koboldt DC, Steinberg KM, Larson DE, Wilson RK, Mardis ER. The next-generation sequencing revolution and its impact on genomics. Cell. 2013;155: 27–38. 10.1016/j.cell.2013.09.006 24074859 PMC3969849

[2] Chiu CY. Viral pathogen discovery. Curr Opin Microbiol. 2013;16: 468–478. 10.1016/j.mib.2013.05.001 23725672 PMC5964995

[3] Wolfe ND, Dunavan CP, Diamond J. Origins of major human infectious diseases. Nature. 2007;447: 279–283. 17507975 10.1038/nature05775 PMC7095142

[4] McMullan LK, Folk SM, Kelly AJ, MacNeil A, Goldsmith CS, Metcalfe MG, et al A new phlebovirus associated with severe febrile illness in Missouri. N Engl J Med. 2012;367: 834–841. 10.1056/NEJMoa1203378 22931317

[5] Briese T, Paweska JT, McMullan LK, Hutchison SK, Street C, Palacios G, et al Genetic detection and characterization of Lujo virus, a new hemorrhagic fever-associated arenavirus from southern Africa. PLoS Pathog. 2009;5 e1000455 10.1371/journal.ppat.1000455 19478873 PMC2680969

[6] Xu B, Liu L, Huang X, Ma H, Zhang Y, Du Y, et al Metagenomic analysis of fever, thrombocytopenia and leukopenia syndrome (FTLS) in Henan Province, China: discovery of a new bunyavirus. PLoS Pathog. 2011;7 e1002369 10.1371/journal.ppat.1002369 22114553 PMC3219706

[7] Yu X-J, Liang M-F, Zhang S-Y, Liu Y, Li J-D, Sun Y-L, et al Fever with thrombocytopenia associated with a novel bunyavirus in China. N Engl J Med. 2011;364: 1523–1532. 10.1056/NEJMoa1010095 21410387 PMC3113718

[8] Van Boheemen S, de Graaf M, Lauber C, Bestebroer TM, Raj VS, Zaki AM, et al Genomic characterization of a newly discovered coronavirus associated with acute respiratory distress syndrome in humans. mBio. 2012;3 pii: e00473–12 10.1128/mBio.00473-12 23170002 PMC3509437

[9] Grard G, Fair JN, Lee D, Slikas E, Steffen I, Muyembe JJ, et al A novel rhabdovirus associated with acute hemorrhagic fever in central Africa. PLoS Pathog. 2012;8 e1002924 10.1371/journal.ppat.1002924 23028323 PMC3460624

[10] Malboeuf CM, Yang X, Charlebois P, Qu J, Berlin AM, Casali M, et al Complete viral RNA genome sequencing of ultra-low copy samples by sequence-independent amplification. Nucleic Acids Res. 2013;41 e13 10.1093/nar/gks794 22962364 PMC3592391

[11] Schmieder R, Edwards R. Quality control and preprocessing of metagenomic datasets. Bioinforma Oxf Engl. 2011;27: 863–864. 10.1093/bioinformatics/btr026 PMC3051327 21278185

[12] Namiki T, Hachiya T, Tanaka H, Sakakibara Y. MetaVelvet: an extension of Velvet assembler to de novo metagenome assembly from short sequence reads. Nucleic Acids Res. 2012;40 10.1093/nar/gks678 PMC3488206 22821567

[13] Grabherr MG, Haas BJ, Yassour M, Levin JZ, Thompson DA, Amit I, et al Full-length transcriptome assembly from RNA-Seq data without a reference genome. Nat Biotechnol. 2011;29: 644–652. 10.1038/nbt.1883 21572440 PMC3571712

[14] Huson DH, Mitra S, Ruscheweyh H-J, Weber N, Schuster SC. Integrative analysis of environmental sequences using MEGAN4. Genome Res. 2011;21: 1552–1560. 10.1101/gr.120618.111 21690186 PMC3166839

[15] Katoh K, Misawa K, Kuma K, Miyata T. MAFFT: a novel method for rapid multiple sequence alignment based on fast Fourier transform. Nucleic Acids Res. 2002;30: 3059–3066. 12136088 10.1093/nar/gkf436 PMC135756

[16] Capella-Gutiérrez S, Silla-Martínez JM, Gabaldón T. trimAl: a tool for automated alignment trimming in large-scale phylogenetic analyses. Bioinforma Oxf Engl. 2009;25: 1972–1973. 10.1093/bioinformatics/btp348 PMC2712344 19505945

[17] Darriba D, Taboada GL, Doallo R, Posada D. ProtTest 3: fast selection of best-fit models of protein evolution. Bioinforma Oxf Engl. 2011;27: 1164–1165. 10.1093/bioinformatics/btr088 PMC5215816 21335321

[18] Dimmic MW, Rest JS, Mindell DP, Goldstein RA. rtREV: an amino acid substitution matrix for inference of retrovirus and reverse transcriptase phylogeny. J Mol Evol. 2002;55: 65–73. 12165843 10.1007/s00239-001-2304-y

[19] Stamatakis A. RAxML-VI-HPC: maximum likelihood-based phylogenetic analyses with thousands of taxa and mixed models. Bioinforma Oxf Engl. 2006;22: 2688–2690. 10.1093/bioinformatics/btl446 16928733

[20] Ronquist F, Huelsenbeck JP. MrBayes 3: Bayesian phylogenetic inference under mixed models. Bioinforma Oxf Engl. 2003;19: 1572–1574. 10.1093/bioinformatics/btg180 12912839

[21] Coffey LL, Page BL, Greninger AL, Herring BL, Russell RC, Doggett SL, et al Enhanced arbovirus surveillance with deep sequencing: Identification of novel rhabdoviruses and bunyaviruses in Australian mosquitoes. Virology. 2014;448: 146–158. 10.1016/j.virol.2013.09.026 24314645 PMC3870893

[22] Branco LM, Grove JN, Boisen ML, Shaffer JG, Goba A, Fullah M, et al Emerging trends in Lassa fever: redefining the role of immunoglobulin M and inflammation in diagnosing acute infection. Virol J. 2011;8: 478 10.1186/1743-422X-8-478 22023795 PMC3223505

[23] Bhattarai N, Stapleton JT. GB virus C: the good boy virus? Trends Microbiol. 2012;20: 124–130. 10.1016/j.tim.2012.01.004 22325031 PMC3477489

[24] Kuzmin IV, Novella IS, Dietzgen RG, Padhi A, Rupprecht CE. The rhabdoviruses: Biodiversity, phylogenetics, and evolution. Infect Genet Evol. 2009;9: 541–553. 10.1016/j.meegid.2009.02.005 19460320

[25] Assenberg R, Delmas O, Morin B, Graham SC, De Lamballerie X, Laubert C, et al Genomics and structure/function studies of Rhabdoviridae proteins involved in replication and transcription. Antiviral Res. 2010;87: 149–161. 10.1016/j.antiviral.2010.02.322 20188763

[26] Gubala A, Davis S, Weir R, Melville L, Cowled C, Boyle D. Tibrogargan and Coastal Plains rhabdoviruses: genomic characterization, evolution of novel genes and seroprevalence in Australian livestock. J Gen Virol. 2011;92: 2160–2170. 10.1099/vir.0.026120-0 21593274

[27] Walker PJ. Bovine ephemeral fever in Australia and the world. Curr Top Microbiol Immunol. 2005;292: 57–80. 15981468 10.1007/3-540-27485-5_4

[28] Blasdell KR, Voysey R, Bulach D, Joubert DA, Tesh RB, Boyle DB, et al Kotonkan and Obodhiang viruses: African ephemeroviruses with large and complex genomes. Virology. 2012;425: 143–153. 10.1016/j.virol.2012.01.004 22305623

[29] Ammar E-D, Tsai C-W, Whitfield AE, Redinbaugh MG, Hogenhout SA. Cellular and molecular aspects of rhabdovirus interactions with insect and plant hosts. Annu Rev Entomol. 2009;54: 447–468. 10.1146/annurev.ento.54.110807.090454 18793103

[30] Fredericks DN, Relman DA. Sequence-based identification of microbial pathogens: a reconsideration of Koch’s postulates. Clin Microbiol Rev. 1996;9: 18–33. 8665474 10.1128/cmr.9.1.18 PMC172879

[31] Lipkin WI. Microbe hunting. Microbiol Mol Biol Rev MMBR. 2010;74: 363–377. 10.1128/MMBR.00007-10 20805403 PMC2937520

[32] Yousaf MZ, Qasim M, Zia S, Khan M ur R, Ashfaq UA, Khan S. Rabies molecular virology, diagnosis, prevention and treatment. Virol J. 2012;9 10.1186/1743-422X-9-50 22348291 PMC3307483

[33] Menghani S, Chikhale R, Raval A, Wadibhasme P, Khedekar P. Chandipura Virus: an emerging tropical pathogen. Acta Trop. 2012;124: 1–14. 10.1016/j.actatropica.2012.06.001 22721825

[34] Steffen I, Liss NM, Schneider BS, Fair JN, Chiu CY, Simmons G. Characterization of the Bas-Congo virus glycoprotein and its function in pseudotyped viruses. J Virol. 2013;87: 9558–9568. 10.1128/JVI.01183-13 23785218 PMC3754090

[35] Schnell MJ, McGettigan JP, Wirblich C, Papaneri A. The cell biology of rabies virus: using stealth to reach the brain. Nat Rev Microbiol. 2010;8: 51–61. 10.1038/nrmicro2260 19946287

[36] Das S, Basu A. Viral infection and neural stem/progenitor cell’s fate: implications in brain development and neurological disorders. Neurochem Int. 2011;59: 357–366. 10.1016/j.neuint.2011.02.020 21354238

[37] Van den Pol AN. Viral infections in the developing and mature brain. Trends Neurosci. 2006;29: 398–406. 16806513 10.1016/j.tins.2006.06.002

