# Supplementary figures and images for: Discovery of Novel Rhabdoviruses in the Blood of Healthy Individuals from West Africa
Source: PLoS Negl Trop Dis. 2015 Mar 17;9(3):e0003631. doi: 10.1371/journal.pntd.0003631 (PMC4363514; doi:10.1371/journal.pntd.0003631)

|                     | Febrile<br>(n=195) | Afebrile<br>(n=328) |
|---------------------|--------------------|---------------------|
| <b>Gender (M/F)</b> | 51/49              | 49/51               |
| <b>Age (median)</b> | 2-79 (33)          | 1-98 (45)           |

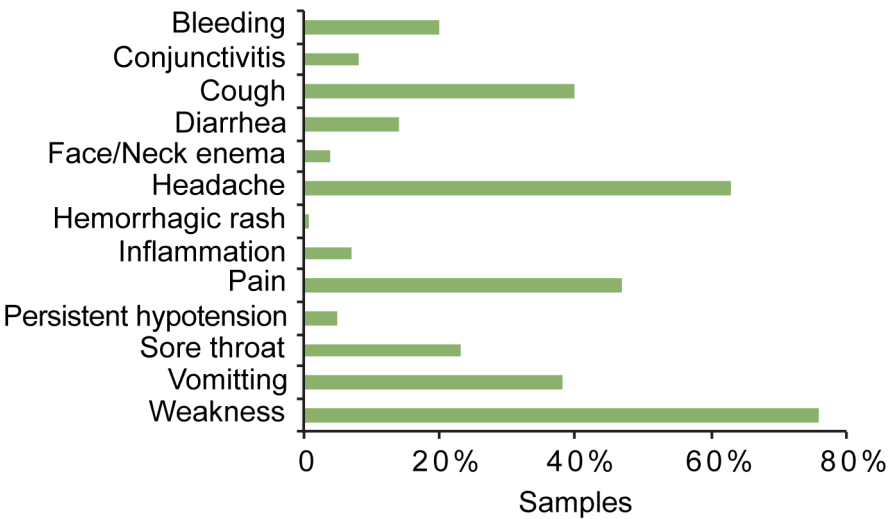

Supplement: S1 Fig — The age and gender of UAFI patients and healthy controls is indicated in the top table. Clinical symptoms of UAFI patients are described in the bar graph. The attending physician observed and recorded symptoms were at the time the patient was admitted to the hospital. (PDF) [file pntd.0003631.s001.pdf]

**A**

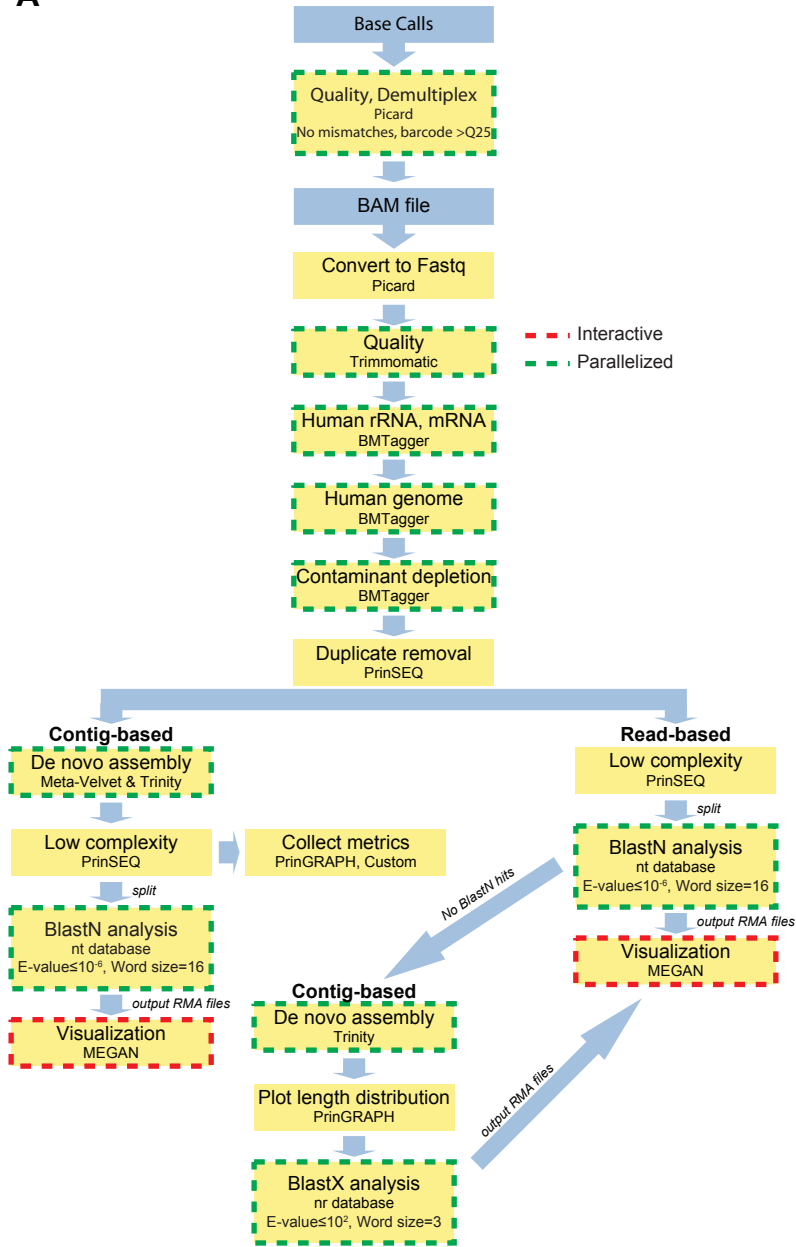

**B**

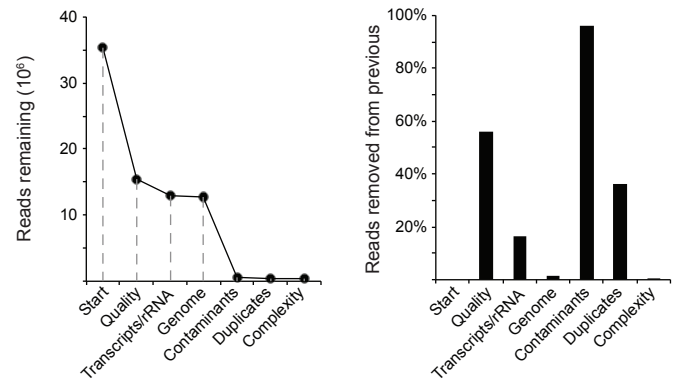

**C**

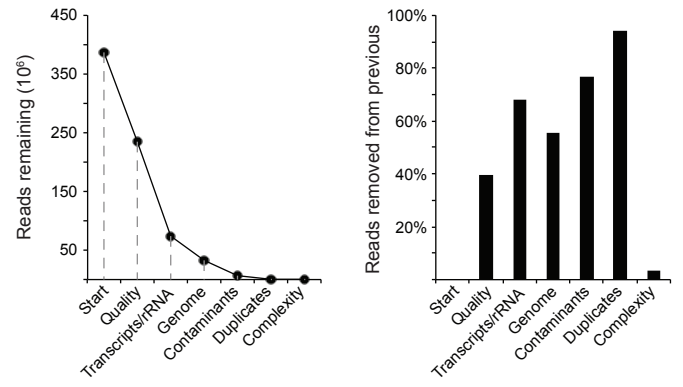

**D**

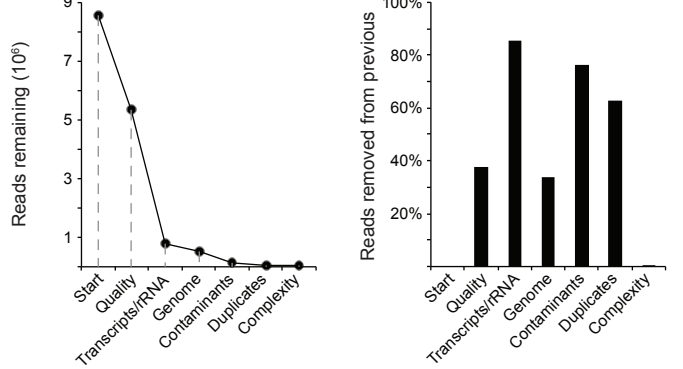

Supplement: S2 Fig — (A) General overview over the various steps in the computational pipeline. (B-D) Plots over the various depletion steps performed in the informatics pipeline from three representative samples containing the EKV rhabdoviruses (left panel = overall reads retained after each step, right panel = % reads removed in each step compared to the previous step). (B) Sample 49CMiSeq (singleton, MiSeq, EKV-2). (C) Sample 49CHiSeq (singleton, HiSeq, EKV-2). (D) Sample HP1_LIB11–18 (pool, MiSeq, EKV-1). (PDF) [file pntd.0003631.s002.pdf]

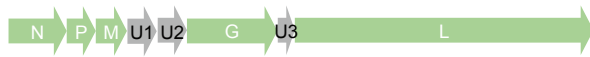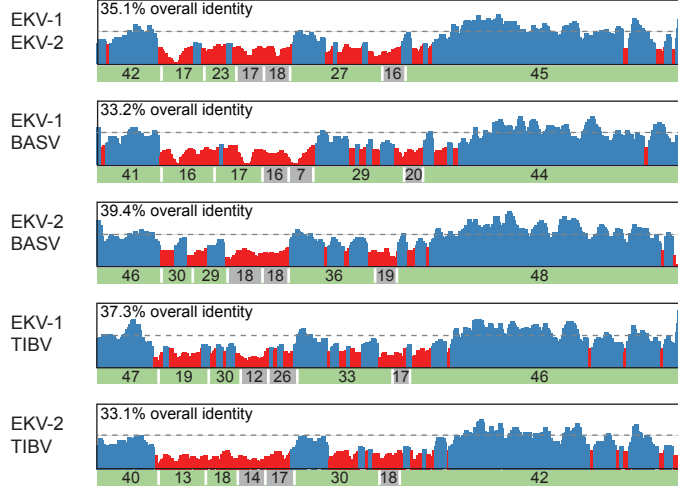

Supplement: S4 Fig — We generated similarity plots by aligning concatenated amino acid sequences and calculating scanning amino acid pairwise identities using a 50 bp window. The x-axis represents the amino acid position along the concatenated rhabdovirus amino acid sequence and the y-axis represent percent pairwise similarity. The percent identity of each pairwise comparison for the individual genes is shown beneath each plot (dashed grey line = 50% identity; red blocks = less than 30% identity). (PDF) [file pntd.0003631.s004.pdf]

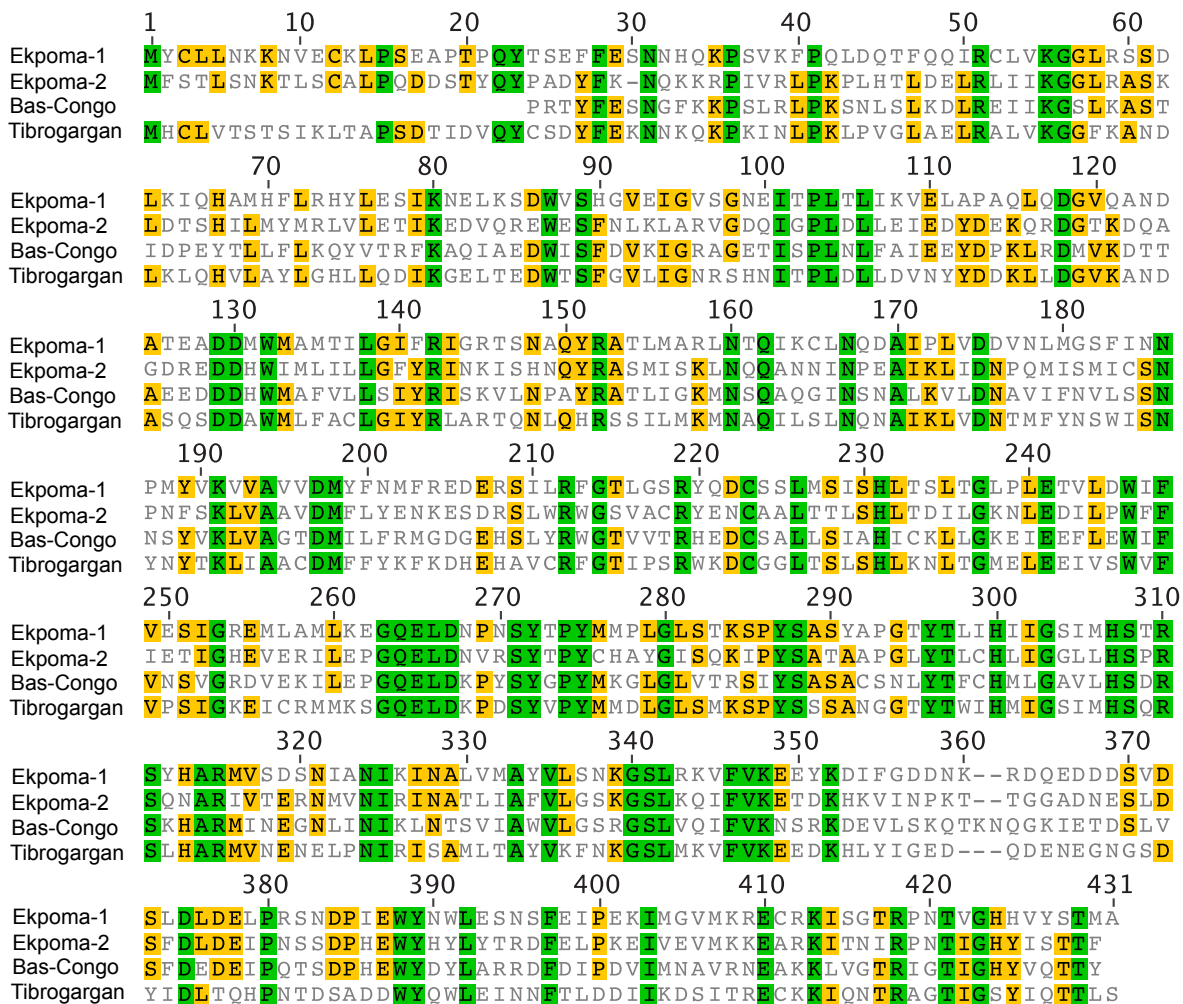

|             | Ekpoma-1 | Ekpoma-2 | Bas-Congo | Tibrogargan |
|-------------|----------|----------|-----------|-------------|
| Ekpoma-1    |          | 41.4%    | 40.3%     | 47.3%       |
| Ekpoma-2    | 41.4%    |          | 45.5%     | 39.5%       |
| Bas-Congo   | 40.3%    | 45.5%    |           | 39.8%       |
| Tibrogargan | 47.3%    | 39.5%    | 39.8%     |             |

Supplement: S5 Fig — We aligned complete nucleoprotein amino acid sequences from the indicated rhabdoviruses using MAFFT. A complete nucleoprotein sequence for BASV is not available. Residues colored green represent identical amino acids in all four viruses; residues colored yellow represent identical amino acids in three of the four viruses. The overall pairwise identity for each set of compared viruses is shown in the table. (PDF) [file pntd.0003631.s005.pdf]

**A**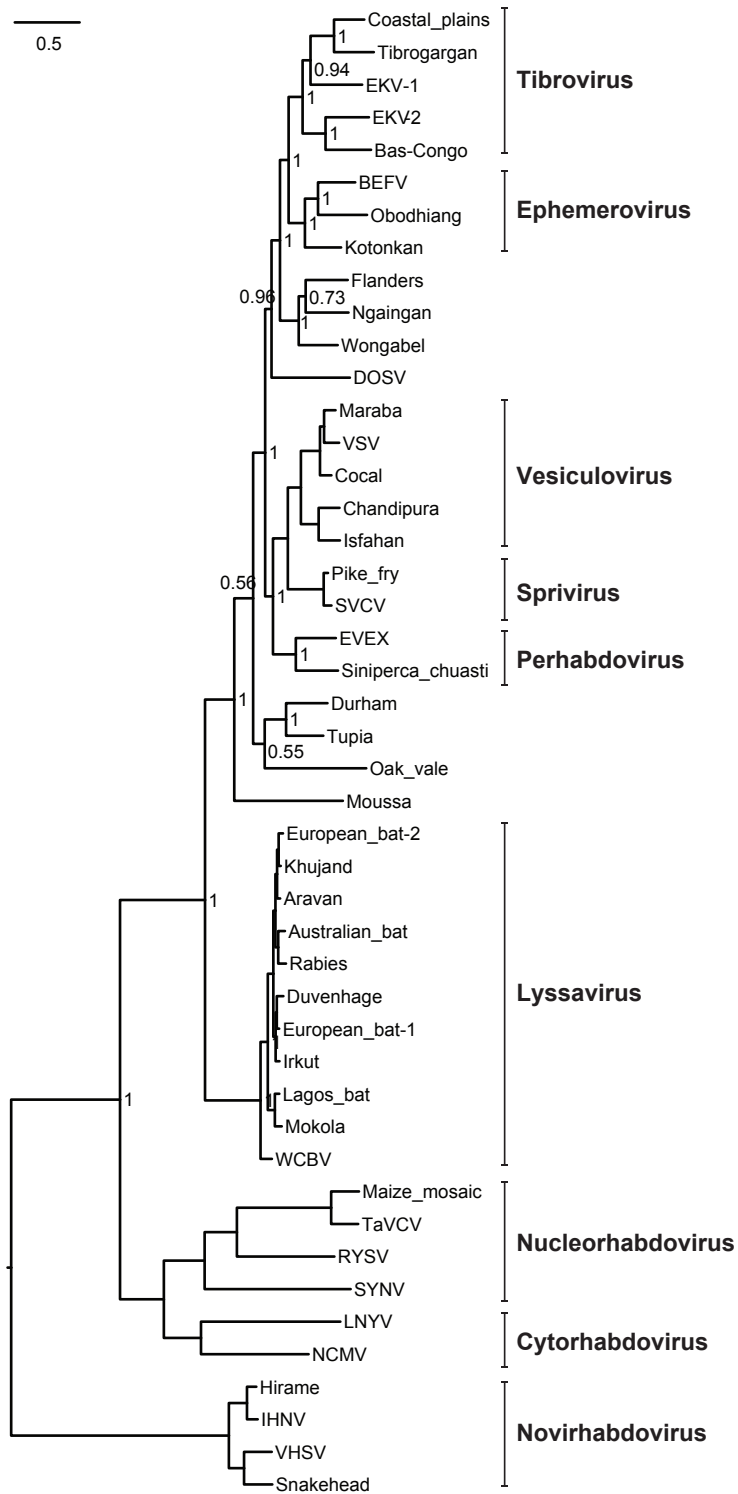**B**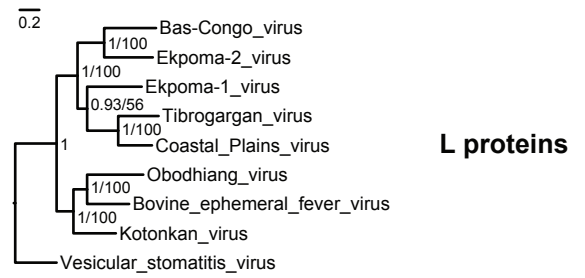**C**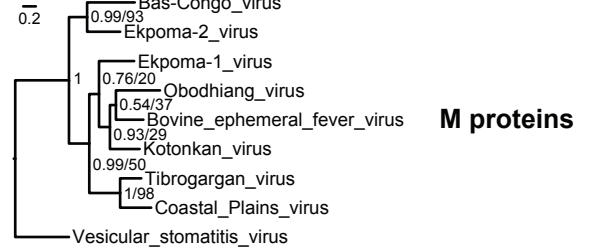**D**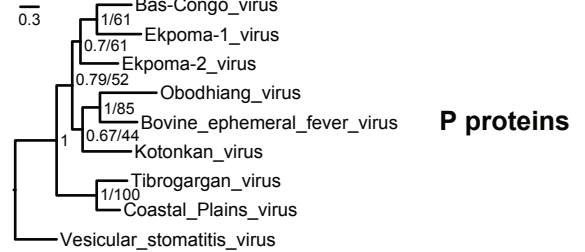**E**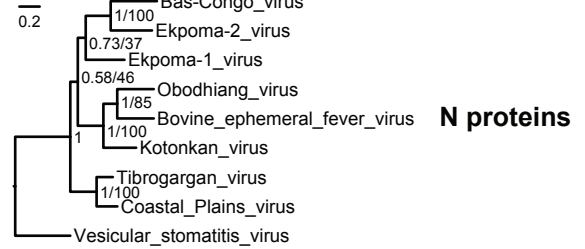**F**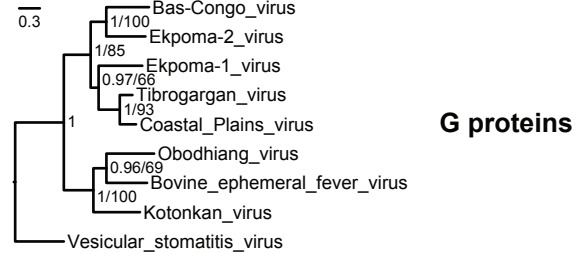

Supplement: S6 Fig — We created Bayesian and maximum likelihood phylogenetic trees using full-length proteins obtained from GenBank. (A) Bayesian tree of full-length polymerase (L) proteins based on alignments from all obtained rhabdovirus sequences. The tree was rooted using the novirhabdovirus clade and posterior support values are shown for key nodes. (B-F) Trees based on alignments of the tibroviruses and ephemeroviruses using vesicular stomatitis virus as an outgroup. (B) L proteins, (C) M proteins, (D) P proteins, (E) N proteins, and (F) G proteins. Bootstrap support values and posterior support are shown for each node (500 pseudo-replicates). Trees were rooted using vesicular stomatitis virus. Scale bar = nucleotide substitutions/site. (PDF) [file pntd.0003631.s006.pdf]

**A**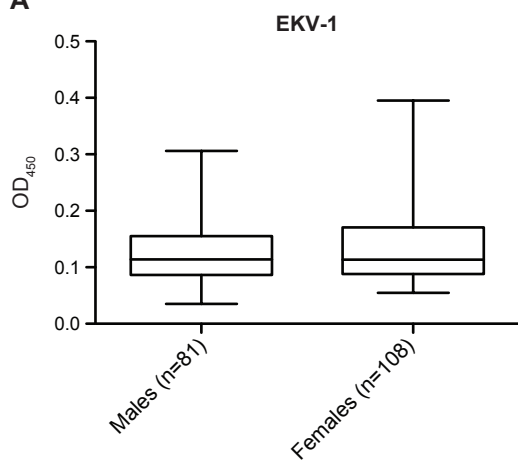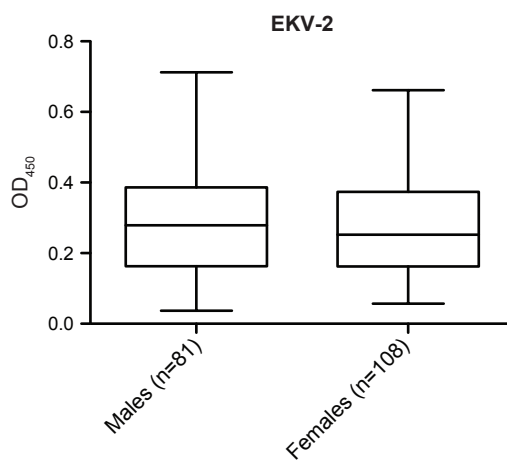**B**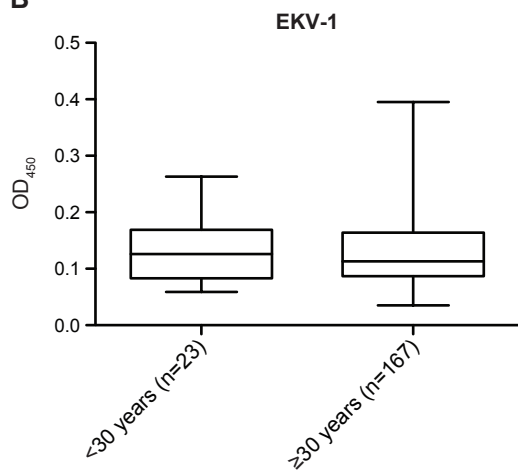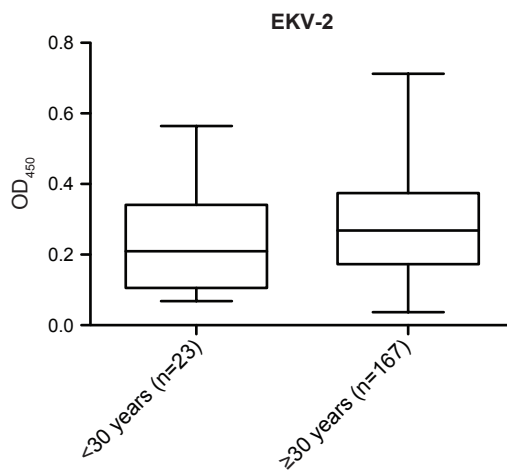

Supplement: S7 Fig — (A, B) Box plots showing the mean and the min to max raw OD450 values obtained from IgG ELISAs specific for EKV-1 and EKV-2. (A) Gender distribution. (B) Samples were grouped into bins of individuals younger than 30 years old or 30 years and older. (A, B) Distributions were compared using a Mann-Whitney test, but no statistical significant differences were observed among the groups. (PDF) [file pntd.0003631.s007.pdf]

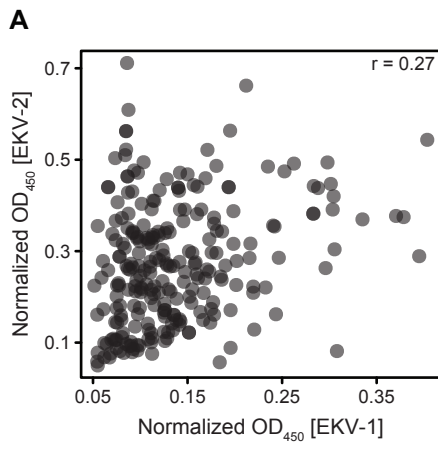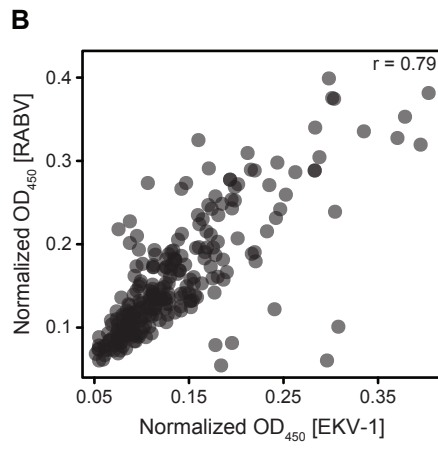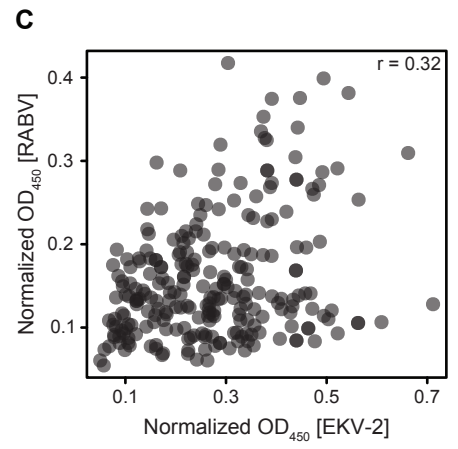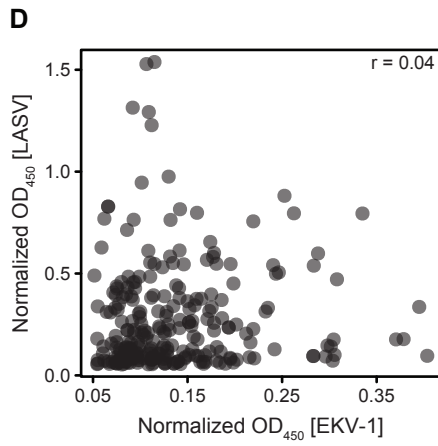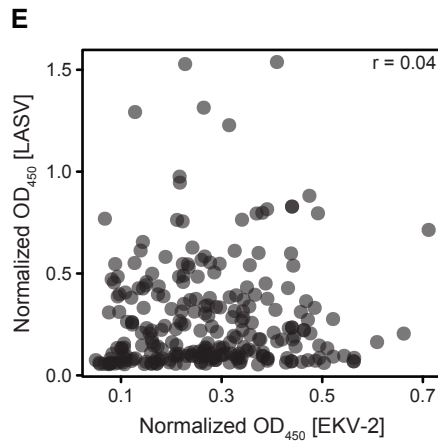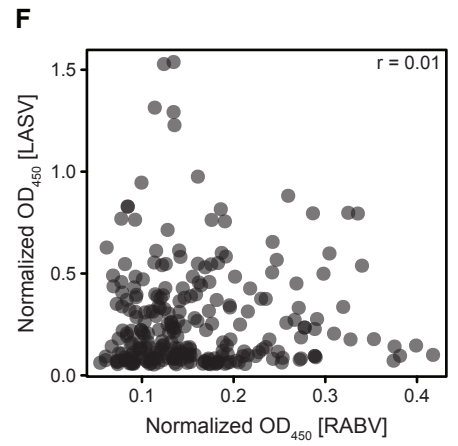

Supplement: S8 Fig — OD450 values obtained from IgG ELISA assays specific for EKV-1, EKV-2, LASV, and rabies virus (RABV) were normalized by comparison to a calibration series run on each plate and plotted against each other. r = Pearson correlation coefficient. (PDF) [file pntd.0003631.s008.pdf]

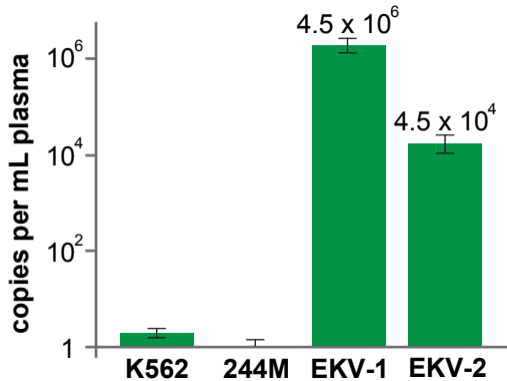

Supplement: S9 Fig — We determined viral copy number using RNA extracted from plasma, primers that target the polymerase (L) gene and serial dilutions of a synthetic amplicon corresponding to the amplified target. We repeated each PCR experiment three times independently. Total human RNA purified from K562 from leukocytes and RNA purified from 244M, the plasma of an afebrile control, were used as a controls. (PDF) [file pntd.0003631.s009.pdf]

**A**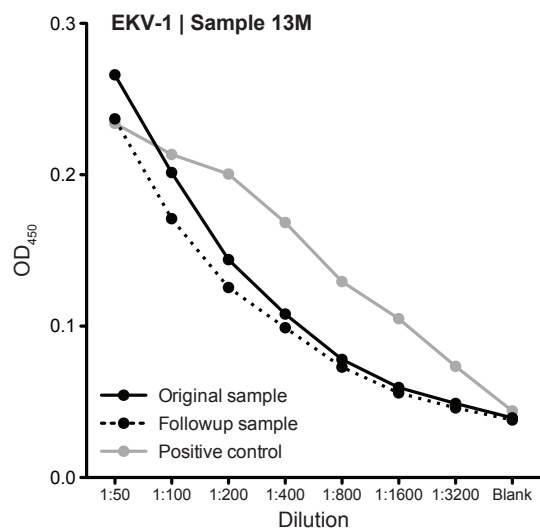**B**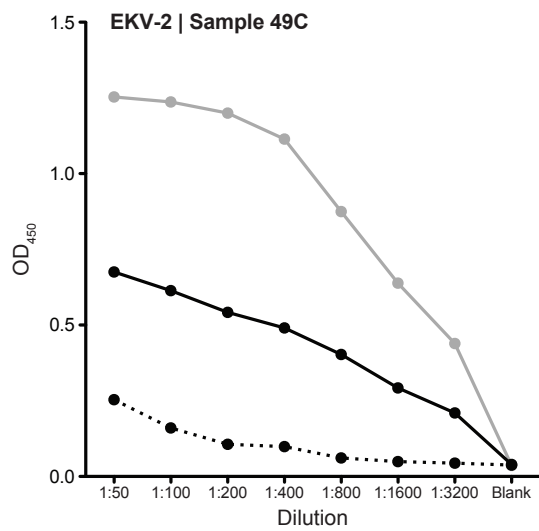

Supplement: S11 Fig — We compared a dilution series of the original sample to the follow-up sample for 13M and 49C with α-His tag IgG as a positive control. (A) Raw OD450 values for samples from patient 13M on EKV-1 NP-coated ELISA plate. (B) Raw OD450 values for samples from patient 49C on EKV-2 NP-coated ELISA plate. (PDF) [file pntd.0003631.s011.pdf]
