# Supplementary material for: Discovery of Novel Rhabdoviruses in the Blood of Healthy Individuals from West Africa
Source: PLoS Negl Trop Dis. 2015 Mar 17;9(3):e0003631. doi: 10.1371/journal.pntd.0003631 (PMC4363514; doi:10.1371/journal.pntd.0003631)
Supplement: S3 Fig — The distributions of the major viruses identified in UAFI patients and healthy individuals were compared using a two-tailed Fisher’s exact test (ns = not significant). LASV was the only virus shown to be significantly enriched in UAFI patients. No viruses were found to be significantly enriched in the healthy individuals. Only the results for LASV, GB virus C and the Ekpoma rhabdoviruses are shown. (PDF) [file pntd.0003631.s003.pdf]

	Positive?		Fisher test	
LASV	**No**	**Yes**	**Odds ratio**	***P*-value**
**UAFI**	16	104	18.47	0.002
**Healthy**	0	58		

GB virus C	No	Yes	Odds ratio	*P*-value
**UAFI**	95	25	0.54	0.097 (ns)
**Healthy**	39	19		

Ekpoma viruses	No	Yes	Odds ratio	*P*-value
**UAFI**	120	0	0.09	0.105 (ns)
**Healthy**	56	2		
