# Supplementary material for: Discovery of Novel Rhabdoviruses in the Blood of Healthy Individuals from West Africa
Source: PLoS Negl Trop Dis. 2015 Mar 17;9(3):e0003631. doi: 10.1371/journal.pntd.0003631 (PMC4363514; doi:10.1371/journal.pntd.0003631)
Supplement: S10 Fig — We performed reverse transcription followed by PCR on RNA extracted from the original plasma samples and follow up plasma samples and electrophoresed on a 2.2% agarose gel with ethidium bromide. Primer sets were specific for either EKV-1 or EKV-2. (PDF) [file pntd.0003631.s010.pdf]

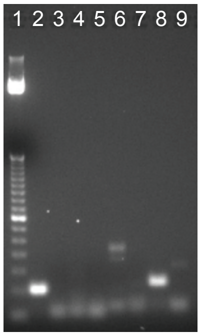

1. Ladder
2. EKV-1 primer set, 13M - original sample
3. EKV-1 primer set, 13M - follow up sample
4. EKV-1 primer set, 49C - original sample
5. EKV-1 primer set, 49C - follow up sample
6. EKV-2 primer set, 13M - original sample
7. EKV-2 primer set, 13M - follow up sample
8. EKV-2 primer set, 49C - original sample
9. EKV-2 primer set, 49C - follow up sample
