# Supplementary material for: Discovery of Novel Rhabdoviruses in the Blood of Healthy Individuals from West Africa
Source: PLoS Negl Trop Dis. 2015 Mar 17;9(3):e0003631. doi: 10.1371/journal.pntd.0003631 (PMC4363514; doi:10.1371/journal.pntd.0003631)
Supplement: S5 Table — (DOCX) [file pntd.0003631.s016.docx]

Table S5: Clinical samples and RNA-seq libraries

| **Sample type** | **Total samples** | **Singleton RNA-seq libraries** | **Pooled RNA-seq libraries** | **Total RNA-seq libraries** |
| --- | --- | --- | --- | --- |
| Febrile | 195 | 94 | 26 | 120 |
| Afebrile | 343 | 34 | 24 | 58 |
