# Supplementary material for: Discovery of Novel Rhabdoviruses in the Blood of Healthy Individuals from West Africa
Source: PLoS Negl Trop Dis. 2015 Mar 17;9(3):e0003631. doi: 10.1371/journal.pntd.0003631 (PMC4363514; doi:10.1371/journal.pntd.0003631)
Supplement: S1 Text — (PDF) [file pntd.0003631.s018.pdf]

Supplementary Text - Additional information about the identified viruses

Human Adenovirus C

We believe that reads from human adenovirus C, which were identified in 96% of the samples (median 34 reads/sample), were contaminants from the library preparation process. We treat all of our samples with DNase; therefore, we should not detect DNA viruses. Furthermore, when the adenoviral reads are realigned to the complete 35.9kb human adenovirus C genome (GenBank accession: NC_001405), the reads only align to a relatively narrow 7.7kb region of the genome from ~10,929 – 18,626. We never identify reads that align to other parts of the genome. Since adenoviruses are widely used as vectors for cloning and protein expression, we think it is likely the adenoviral reads come from contamination during the library preparation process. We have not attempted to amplify human adenovirus C directly from our plasma samples.

Dengue Virus

We identified one sample with dengue virus. This sample only contained 8 paired-end reads. We attempted to PCR dengue directly from the patient's plasma, but no amplification product could be detected. We also attempted to realign the reads from this sample to multiple full-length dengue virus genomes to determine if there were additional reads present in the sample. However, we did not find any additional reads. Dengue virus RNA copies can fall precipitously after the acute phase even though the patient remains ill. Therefore, it is possible our attempts to detect dengue were too late. This situation highlights the limits of using nucleic acids to detect viral infections, even very sensitive methods such as next-generation sequencing. Serological assays are needed to provide further evidence this patient was actually infected with dengue virus.

Lassa Fever

The significant number of Lassa-positive samples is not surprising because our study was conducted in a Lassa-endemic region; however, we note that all of the samples were pre-screened specifically for Lassa. Thus, we analyzed the Lassa diagnostic primer binding site in several samples to determine if divergent Lassa variants were escaping amplification. We found no evidence of polymorphisms in the primer binding site that would prevent detection by the conventional PCR assay used at ISTH. It is possible that the Lassa reads identified in our libraries are contaminants from other samples processed in the same laboratory (whether in Nigeria or the U.S.). The median number of Lassa reads in the RNA-seq libraries is only 30. Only one library contained more than 1,000 Lassa reads. The number of Lassa reads is lower than usually observed in infected patients.

Hepatitis B

One sample (602) contained 4,760 hepatitis B reads and 6,425 HIV-1 reads. It is possible that in this individual we detected Hepatitis B transcripts—not the DNA genome. Although we use centrifugation to separate the buffy coat from the plasma,

some of the infected cells in this sample may have lysed. The released RNA remained in the plasma fraction and was transcribed into cDNA as part of the library construction process.

Single-Stranded RNA Viruses

We discovered several single-stranded RNA viruses associated with marine environments and plants. These viruses included bacillariornaviruses, dicistroviruses, labyrnaviruses, marine JB-like viruses, ourmiaviruses, plasmoparaviruses, and tombusviruses. In each case, the reads we identified were less than 50% similar at the amino acid level to the next closest match in GenBank. Thus, we believe that these particular viruses have not been previously described. We were able to assemble a complete, or nearly complete, genome of the novel bacillariornavirus. The assembled genome was 9.3 kb and the overall homology less than 40% identical to bacillariornavirus sequences deposited in GenBank. We detected reads from small single-stranded RNA viruses in many samples—mostly UAFI patients but also some afebrile controls (we detected ≥5 bacillariornavirus reads in 65 different libraries). One sample contained more than 15,000 bacillariornavirus reads (median 198 reads/sample). Because these viruses appear in both UAFI and afebrile controls, we think they are likely contaminants. Also, some of these viruses resemble ubiquitous picornaviruses often found in aquatic or other environmental samples. These viruses could have been introduced into the samples if dust, dirt or contaminated water at one of the processing steps.

Ekpoma-1 and -2

Ekpoma-1 was discovered in a pool of 15 afebrile individuals (sample HP1_LIB11-18) and Ekpoma-2 discovered in a pool of 16 individuals (sample DFultra2). In both cases reads similar to rhabdovirus sequences deposited in GenBank were identified through BLASTx searches.
